# Nomenclatural issues concerning cultured yeasts and other fungi: why it is important to avoid unneeded name changes

**DOI:** 10.1186/s43008-021-00067-x

**Published:** 2021-07-13

**Authors:** Andrey Yurkov, Artur Alves, Feng-Yan Bai, Kyria Boundy-Mills, Pietro Buzzini, Neža Čadež, Gianluigi Cardinali, Serge Casaregola, Vishnu Chaturvedi, Valérie Collin, Jack W. Fell, Victoria Girard, Marizeth Groenewald, Ferry Hagen, Chris Todd Hittinger, Aleksey V. Kachalkin, Markus Kostrzewa, Vassili Kouvelis, Diego Libkind, Xinzhan Liu, Thomas Maier, Wieland Meyer, Gábor Péter, Marcin Piątek, Vincent Robert, Carlos A. Rosa, Jose Paulo Sampaio, Matthias Sipiczki, Marc Stadler, Takashi Sugita, Junta Sugiyama, Hiroshi Takagi, Masako Takashima, Benedetta Turchetti, Qi-Ming Wang, Teun Boekhout

**Affiliations:** 1 Leibniz Institute DSMZ-German Collection of Microorganisms and Cell Cultures, Inhoffenstrasse 7B, 38124 Braunschweig, Germany; 2 Departamento de Biologia, CESAM - Centro de Estudos do Ambiente e do Mar, Universidade de Aveiro, 3810-193 Aveiro, Portugal; 3 State Key Laboratory of Mycology, Institute of Microbiology, Chinese Academy of Sciences, No. 3-1 Beichen West Road., Chaoyang District, Beijing, 100101 People’s Republic of China; 4 Department of Food Science and Technology, Phaff Yeast Culture Collection, University of California, Davis, One Shields Avenue, Davis, CA 95616 USA; 5 Department of Agricultural, Food and Environmental Sciences & Industrial Yeasts Collection DBVPG, University of Perugia, Borgo XX Giugno 74, 06121 Perugia, Italy; 6 Department of Food Science and Technology, Biotechnical Faculty, University of Ljubljana, Jamnikarjeva ul. 101, 1000 Ljubljana, Slovenia; 7 Department of Pharmaceutical Sciences, University of Perugia, Borgo XX Giugno 74, 06121 Perugia, Italy; 8 Micalis Institute, INRA, AgroParisTech, CIRM-Levures, Université Paris-Saclay, 78350 Jouy-en-Josas, France; 9 Mycology Laboratory, Wadsworth Center, New York State Department of Health, 120 New Scotland Avenue, Albany, NY 12208 USA; 10 BioMérieux, R&D Microbiologie, Route de Port Michaud, 38390 La Balme les Grottes, France; 11 Emeritus Professor, Marine Biology and Ecology, Rosenstiel School of Marine and Atmospheric Science, University of Miami, 4600 Rickenbacker Causeway, Key Biscayne, FL 33149 USA; 12 Westerdijk Fungal Biodiversity Institute, Uppsalalaan 8, 3584CT, Utrecht, The Netherlands; 13 Laboratory of Genetics, Wisconsin Energy Institute, DOE Great Lakes Bioenergy Research Center, Center for Genomic Science Innovation, J. F. Crow Institute for the Study of Evolution, University of Wisconsin-Madison, 1552 University Avenue, Madison, WI 53726-4084 USA; 14 Faculty of Soil Science, Lomonosov Moscow State University, Leninskie Gory 1-12, 119991 Moscow, Russia; 15 All-Russian Collection of Microorganisms (VKM), Skryabin Institute of Biochemistry and Physiology of Microorganisms (IBPM RAS), Russian Academy of Sciences, Prospect Nauki 5, 142290 Puschino, Russia; 16 Bruker Daltonik GmbH, Fahrenheitstraße 4, 28359 Bremen, Germany; 17 Department of Genetics and Biotechnology, Faculty of Biology, National and Kapodistrian University of Athens, Panepistemiopolis, 15701 Athens, Greece; 18 Centro de Referencia en Levaduras y Tecnología Cervecera (CRELTEC), Instituto Andino Patagónico de Tecnologías Biológicas y Geoambientales (IPATEC) CONICET - Universidad Nacional del Comahue, Quintral 1250, San Carlos de Bariloche, Rio Negro Argentina; 19 Molecular Mycology Research Laboratory, Center for Infectious Diseases and Microbiology, Westmead Clinical School, Sydney Medical School, Faculty of Medicine and Health, The University of Sydney, Camperdown, NSW 2006 Australia; 20 Marie Bashir Institute for Infectious Diseases and Biosecurity, The University of Sydney, Camperdown, NSW 2006 Australia; 21 Westmead Institute for Medical Research, 176 Hawkesbury Rd, Westmead, NSW 2145 Australia; 22 Westmead Hospital (Research and Education Network), Darcy Rd, Westmead, NSW 2145 Australia; 23 National Collection of Agricultural and Industrial Microorganisms, Institute of Food Science and Technology, Hungarian University of Agriculture and Life Sciences, Somlói út 14-16, Budapest, H-1118 Hungary; 24 Department of Mycology, W. Szafer Institute of Botany of the Polish Academy of Sciences, Lubicz ul. 46, 31-512 Kraków, Poland; 25 Departamento de Microbiologia, Instituto de Ciências Biológicas, Universidade Federal de Minas Gerais, Avenida Antônio Carlos, 6627 Pampulha, Belo Horizonte, MG 31270-901 Brazil; 26 UCIBIO, Departamento de Ciências da Vida, Faculdade de Ciências e Tecnologia, Universidade Nova de Lisboa, Largo da Torre, 2825-149 Caparica, Portugal; 27 Departamento de Ciências da Vida, PYCC - Portuguese Yeast Culture Collection, Faculdade de Ciências e Tecnologia, Universidade Nova de Lisboa, Largo da Torre, 2825-149 Caparica, Portugal; 28 Department of Genetics and Applied Microbiology, University of Debrecen, Egyetem tér 1, Debrecen, 4010 Hungary; 29 Department Microbial Drugs, Helmholtz Centre for Infection Research, and German Centre for Infection Research (DZIF), partner site Hannover-Braunschweig, Inhoffenstrasse 7, 38124 Braunschweig, Germany; 30 Department of Microbiology, Meiji Pharmaceutical University, 2 Chome-522-1 Noshio, Kiyose, Tokyo, 204-8588 Japan; 31 Department of Botany, National Museum of Nature and Science, 4-1-1 Amakubo, Tsukuba, Ibaraki 305-0005 Japan; 32 TechnoSuruga Laboratory Co., Ltd., 388-1, Nagasaki, Shimuzu, Shizuoka 424-0065 Japan; 33 Division of Biological Science, Graduate School of Science and Technology, Nara Institute of Science and Technology, 8916-5 Takayama, Ikoma, Nara, 630-0192 Japan; 34 Laboratory of Yeast Systematics, Research Institute for Agricultural and Life Sciences, Tokyo University of Agriculture, 1-1-1 Sakuragaoka, Setagaya, Tokyo, 156-8502 Japan; 35 College of Life Sciences, Hebei University, 180 Wusi Dong Road, Lian Chi District, Baoding City, Hebei Province 071002 People’s Republic of China; 36 Institute of Biodiversity and Ecosystem Dynamics (IBED), University of Amsterdam, 904 Science Park, 1098 XH Amsterdam, The Netherlands

**Keywords:** Typification, Nomenclatural type, Culture collection, Metabolically inactive, Viable strains

## Abstract

The unambiguous application of fungal names is important to communicate scientific findings. Names are critical for (clinical) diagnostics, legal compliance, and regulatory controls, such as biosafety, food security, quarantine regulations, and industrial applications. Consequently, the stability of the taxonomic system and the traceability of nomenclatural changes is crucial for a broad range of users and taxonomists. The unambiguous application of names is assured by the preservation of nomenclatural history and the physical organisms representing a name. Fungi are extremely diverse in terms of ecology, lifestyle, and methods of study. Predominantly unicellular fungi known as yeasts are usually investigated as living cultures. Methods to characterize yeasts include physiological (growth) tests and experiments to induce a sexual morph; both methods require viable cultures. Thus, the preservation and availability of viable reference cultures are important, and cultures representing reference material are cited in species descriptions. Historical surveys revealed drawbacks and inconsistencies between past practices and modern requirements as stated in the *International Code of Nomenclature for Algae, Fungi, and Plants* (ICNafp). Improper typification of yeasts is a common problem, resulting in a large number invalid yeast species names. With this opinion letter, we address the problem that culturable microorganisms, notably some fungi and algae, require specific provisions under the ICNafp. We use yeasts as a prominent example of fungi known from cultures. But viable type material is important not only for yeasts, but also for other cultivable *Fungi* that are characterized by particular morphological structures (a specific type of spores), growth properties, and secondary metabolites. We summarize potential proposals which, in our opinion, will improve the stability of fungal names, in particular by protecting those names for which the reference material can be traced back to the original isolate.

## INTRODUCTION

The *International Code of Nomenclature for algae, fungi, and plants* (ICNafp or the *Code*) represents a set of rules and recommendations regarding the naming of organisms “traditionally treated as algae, fungi, or plants” (Turland et al. 2018). The rules governing fungal nomenclature have undergone major changes, reflecting the diversity of organisms treated as fungi and the methods used to study them. Among most tangible changes, (1) the *Code* adopted the ‘One fungus, one name’ principle in 2011 (Melbourne Code; McNeill et al. 2012), (2) required registration of newly proposed fungal names in a recognized electronic repository in order to be validly published after 2013 (McNeill et al. 2012), and (3) required a statement when the nomenclatural type is a culture that it is preserved in a metabolically inactive state for names published after 2018 (Shenzhen Code; Turland et al. 2018). The modifications to the *Code* are a matter of ongoing discussions in the International Commission on the Taxonomy of Fungi (ICTF) and its sub-commissions and working groups dealing with different groups of fungi. From 2017, decisions to change the requirements for the rules relating only to fungal organisms are now dealt with at Nomenclature Sessions held at International Mycological Congresses (IMCs), and the first changes made by this route were taken at the IMC11 in Puerto Rico in 2018 (May et al. 2019). IMCs now also appoint the Nomenclature Committee for Fungi (NCF), which rules on proposals to conserve or reject names, as well as giving opinions of any proposals made to formally change any rules relating to the naming of fungal organisms.

Organisms regarded as yeasts are phylogenetically diverse and occur in the *Ascomycota* and the *Basidiomycota*. Besides the well-known ascomycetous yeasts in the *Saccharomycotina* and *Taphrinomycotina*, other yeasts are taxonomically related to jelly fungi (*Tremellomycetes*, *Agaricomycotina*), anther smuts (*Microbotryomycetes*, *Pucciniomycotina*), and true smuts (*Ustilaginomycotina*). The taxonomy of basidiomycetous yeasts developed independently from that of the other taxa in those groups (filamentous fungi) and was based on physiological growth profiles, biochemical features of the cell wall, and DNA features, such as molar percentage of guanine + cytosine of the DNA and molecular phylogenetic inferences. For a long time, the taxonomy of filamentous macroscopic and sporophore-forming *Ascomycota* and *Basidiomycota* was largely based on morphology. However, application of molecular systematics and comparative genomics has unified the taxonomies of “yeast” and “filamentous” ascomycetes and basidiomycetes (Selbmann et al. 2014; Liu et al. 2015; Wang et al. 2015a, b, c; Shen et al. 2018; Li et al. 2020; Lücking et al. 2020). Such taxonomic overlap requires careful consideration of history, practice, and taxonomic priority when dealing with names of fungi. Taxonomists of the International Commission on Yeasts (ICY), and editors and authors of the latest and past editions of the monographic book ‘*The Yeasts: A taxonomic study*’ (Kurtzman et al. 2011) and the on-line database www.theyeasts.org (Boekhout et al. 2020) have established a modern framework that is used to provide descriptions of newly proposed yeast taxa.

The number of currently accepted yeast species exceeds 1500 (Kurtzman et al. 2011), increases by about 5% annually (Yurkov 2017) and is soon to reach > 2300 species according to www.theyeasts.org (Boekhout et al. 2020). Novel yeast taxa are mainly published in a few general microbiological journals, such as *International Journal of Systematic and Evolutionary Microbiology* (IJSEM) and *Antonie van Leeuwenhoek*; followed by dedicated yeasts journals, such as *FEMS Yeast Research* or *Yeast*; and several other general, mycological and microbiological journals, such as *Extremophiles, Journal of Clinical Microbiology, Journal of General and Applied Microbiology, Mycopathologia, Mycoscience, Mycological Progress*, *Studies in Mycology*, *Persoonia*, and *PLoS One*.

The ongoing unification of the taxonomy of yeasts and filamentous fungal taxa, and the application of the ‘One fungus, one name’ principle to fungi known as yeasts was the result of intensive discussions leading to well-weighted compromises. Several practices, rules and recommendations were adopted by the yeast community from the field of bacteriology due to the microbial nature of yeasts. Historical surveys revealed drawbacks and inconsistencies between past practices and modern requirements of the ICNafp. Some inconsistencies have been corrected to ensure that names of biotechnological and clinically important yeasts are legitimate and validly published (Kwon-Chung et al. 2002; Kurtzman and Robnett 2003; Kurtzman et al. 2008; Daniel et al. 2014). A few others are a matter of an ongoing fruitful exchange between taxonomists in the NCF/ICTF Nomenclatural Working Groups, including the ICY. One of probably most important taxonomic discussions concerns improper typification of fungi commonly known as yeasts, although the problem is not limited to yeasts (Kijpornyongpan and Aime 2017; Aime et al. 2021).

The problem of potentially invalid names, which may affect approximately 300 names of yeasts, is a threat to the stability of the taxonomy of fungi. A few names of yeasts have been validated already, including the genus *Jaminaea* and the two species *J. angkorensis* and *J. lanaiensis* made by Kijpornyongpan and Aime (2017). The publication created a precedent, implying that many known and used names of yeasts could be argued to be invalid because the requirements of the *Code* had not been properly interpreted. The potentially negative impact of that publication was quickly recognized and repeatedly lamented by yeast researchers during scientific meetings, such as the International Mycological Congress (IMC11 in 2018) and International Specialised Symposia on Yeasts (ISSY33 and ISSY34 in 2017 and 2018, respectively). Another validation of 19 names of basidiomycetous yeasts from the genera *Acaromyces*, *Farysizyma*, and *Meira*, and several other species was quite recently published by Denchev and Denchev (2021). The recent validation additionally strengthened the concern of the yeast community on uncontrolled taxonomic interventions bypassing the existing system of the ICTF Nomenclatural Working Groups. However, the two taxonomic proposals also pointed again to the important old problem that culturable microorganisms, notably many fungi and algae, require more specific and clearer special provisions under the ICNafp. We agree that not all past descriptions of yeast species were made in full accordance with the *Code* in force, and we recognize the confusion generated by the two validations to explain reasons for using a different practice for the designation of type material of yeasts. We emphasize that a careful examination of the concept of typification as depicted in the *Code* is as important as the wording used in the *Code*. We use this article to approach the broader mycological community and to share our views on the further development of the *Code* to better implement the use of living cultures as type material.

### The formal clause

The proposal by Kijpornyongpan and Aime (2017) pointed to the two provisions of the *Code* that account for the majority of potentially invalid names of yeasts (see also Aime et al. 2021). In their proposal, Kijpornyongpan and Aime (2017) stated that the genus *Jaminaea* and the two species names *J. angkorensis* and *J. lanaiensis* were invalid according to Art. 8.4 and 40.7 in ICNafp (Melbourne Code; McNeill et al. 2012) (Table 1). Similarly, Denchev and Denchev (2021) concluded the genera *Acaromyces*, *Farysizyma*, and *Meira* are invalid because their type species are invalid according to Art 40.7 in ICNafp (Shenzhen Code; Turland et al. 2018).

**Table 1:** List of the debated articles, examples, notes and recommendations of the *International Code of Nomenclature for algae, fungi, and plants* (ICNafp 2017, Shenzhen Code; Turland et al. 2018), with selected key points to note in bold

**Art. 8.4.** Type specimens of names of taxa must be preserved permanently and may not be living organisms or cultures. Nevertheless, cultures of algae and fungi, if preserved in a metabolically inactive state (e.g. by lyophilization or deep-freezing to remain alive in that inactive state), are acceptable as types (see also Art. 40.8). **Ex. 11.** “*Dendrobium sibuyanense*” (Lubag-Arquiza & al. in Philipp. Agric. Sci. 88: 484–488. 2005) was described with the statement “Type specimen is a living specimen being maintained at the Orchid Nursery, Department of Horticulture, University of the Philippines Los Baños (UPLB). Collectors: Orville C. Baldos & Ramil R. Marasigan, April 5, 2004”. However, this is a living collection and, as such, is not acceptable as a type. Consequently no type was indicated and the name was not validly published (Art. 40.1). **Ex. 12.** The strain CBS 7351 is acceptable as the type of the name *Candida populi* Hagler & al. (in Int. J. Syst. Bacteriol. 39: 98. 1989) because it is permanently preserved in a metabolically inactive state by lyophilization (see also Rec. 8B.2). **Rec. 8B.2.** In cases where the type of a name is a culture permanently preserved in a metabolically inactive state (see Art. 8.4), any living isolates obtained from it should be referred to as “ex-type” (ex typo), “ex-holotype” (ex holotypo), “ex-isotype” (ex isotypo), etc., in order to make it clear they are derived from the type but are not themselves the nomenclatural type.	
**Art. 9.2.** If a designation of holotype made in the protologue of the name of a taxon is later found to contain errors (e.g. in locality, date, collector, collecting number, herbarium code, specimen identifier, or citation of an illustration), these errors are to be corrected provided that the intent of the original author(s) is not changed. However, **omissions of required information under Art. 40.6–40.8 are not correctable**. **Rec. 9B.1.** In selecting a neotype, particular care and critical knowledge should be exercised because there is usually no guide except personal judgement as to what best fits the protologue; if this selection proves to be faulty it may result in further change.	
**Art. 40.6.** For the name of a new taxon at the rank of genus or below published on or after 1 January 1990, indication of the type **must include one of the words “typus” or “holotypus”, or its abbreviation, or its equivalent in a modern language** (see also Rec. 40A.1 and 40A.4). But in the case of the name of a monotypic (as defined in Art. 38.6) new genus or subdivision of a genus with the simultaneously published name of a new species, indication of the type of the species name is sufficient. **Ex. 7.** When Stephenson described “*Sedum mucizonia* (Ortega) Raym.-Hamet subsp. *urceolatum*” (in Cact. Succ. J. (Los Angeles) 64: 234. 1992) the name was not validly published because the protologue lacked the indication “typus” or “holotypus”, or its abbreviation, or its equivalent in a modern language, a requirement for names published on or after 1 January 1990.	
**Art. 40.7.** For the name of a new species or infraspecific taxon published on or after 1 January 1990 of which the type is a specimen or unpublished illustration, **the single herbarium, collection, or institution in which the type is conserved must be specified** (see also Rec. 40A.5 and 40A.6). **Ex. 8.** In the protologue of *Setaria excurrens* var. *leviflora* Keng ex S. L. Chen (in Bull. Nanjing Bot. Gard. 1988–1989: 3. 1990) the gathering Guangxi Team 4088 was indicated as “模式” [type] and the herbarium where the type is conserved was specified as “中国科学院植物研究所标本室” [Herbarium, Institute of Botany, The Chinese Academy of Sciences], i.e. PE. **Note 4.** Specification of the herbarium, collection, or institution may be made in an abbreviated form, e.g. as given in Index Herbariorum (http://sweetgum.nybg.org/science/ih/) or in the *World directory of collections of cultures of microorganisms* ^a^. **Ex. 9.** When ‘t Hart described “*Sedum eriocarpum* subsp. *spathulifolium*” (in Ot Sist. Bot. Dergisi 2 (2): 7. 1995) the name was not validly published because no herbarium, collection, or institution in which the holotype specimen was conserved was specified. Valid publication was effected when ‘t Hart (in Strid & Tan, Fl. Hellen. 2: 325. 2002) wrote “Type … ‘t Hart HRT-27104 … (U)” while providing a full and direct reference to his previously published Latin diagnosis (Art. 33.1).	
**Art. 40.8.** For the name of a new species or infraspecific taxon **published on or after 1 January 2019** of which the type is a culture, **the protologue must include a statement that the culture is preserved in a metabolically inactive state**.	

^a^ see Culture Collections Information Worldwide of the World Federation for Culture Collections and World Data Center for Microorganisms http://www.wfcc.info/ccinfo/ (CCINFO)

The formal reason is the so-called ‘metabolically inactive’ clause, which stipulates that type specimens of names of taxa must be preserved permanently and may not be living organisms or cultures. Because the descriptions of *J. angkorensis* and *J. lanaiensis* only referred to type strains and did not explicitly specify ‘metabolically inactive’ cultures, Kijpornyongpan and Aime (2017) concluded that the two names were invalid according to the Art. 8.4, ICNafp Melbourne Code (Table 1). It was not, however, appreciated by those authors that a requirement to state that strains were metabolically inactive was mandatory only after the start of 2019 (ICNafp Shenzhen Code; Turland et al. 2018). The non-inclusion of a requirement to make that statement in earlier editions of the *Code*, however, was deliberate (see below).

Additionally, Art. 40.7 states that “*For the name of a new species or infraspecific taxon published on or after 1 January 1990 of which the type is a specimen or unpublished illustration, the single herbarium, collection, or institution in which the type is conserved must be specified.*” Thus, a name is invalid when no herbarium or collection was specified in the description or when several collection numbers are provided in the protologue, as defined in Table 2.

**Table 2:** Condensed glossary of some definition of terms of the ICNapf used in the manuscript

**basionym**	The legitimate, previously published name on which a new combination or name at new rank is based. The basionym does not itself have a basionym; it provides the final epithet, name, or stem of the new combination or name at new rank (**Art. 6.10**).
**epitype**	A specimen or illustration selected to serve as an interpretative type when the holotype, lectotype, or previously designated neotype, or all original material associated with a validly published name, cannot be identified for the purpose of the precise application of the name to a taxon (**Art. 9.9**).
**ex-type (ex typo), ex-holotype (ex holotypo), ex-isotype (ex isotypo), etc.**	A living isolate obtained from the type of a name when this is a culture permanently preserved in a metabolically inactive state (**Rec. 8B.2**).
**holotype**	The one specimen or illustration indicated as the nomenclatural type by the author(s) of a name of a new species or infraspecific taxon or, when no type was indicated, used by the author(s) when preparing the account of the new taxon (**Art. 9.1**, **Note 1**; see also **Art. 9.2**).
**isotype**	A duplicate specimen of the holotype (**Art. 9.5**).
**lectotype**	One specimen or illustration designated from the original material as the nomenclatural type, in conformity with **Art. 9.11** and **9.12**, if the name was published without a holotype, or if the holotype is lost or destroyed, or if a type is found to belong to more than one taxon (**Art. 9.3**). Note: this applies only to names published before 1st January 1990 (**Art. 40.7**).
**neotype**	A specimen or illustration selected to serve as nomenclatural type if no original material is extant or as long as it is missing (**Art. 9.8** and **9.13**; see also **Art. 9.16** and **9.19**).
**paratype**	Any specimen cited in the protologue that is neither the holotype nor an isotype, nor one of the syntypes if in the protologue two or more specimens were simultaneously designated as types (**Art. 9.7**).
**protologue**	Everything associated with a name at its valid publication, e.g. description, diagnosis, illustrations, references, synonymy, geographical data, citation of specimens, discussion, and comments (**Art. 6.13 footnote**).

## HISTORICAL REVIEW

### Provisions of the *Code*

That no living material could be a type has always been a pillar of the *International Code of Botanical Nomenclature* (ICBN), which originally covered also bacteria and viruses (Buchanan 1959). A tentative *Code of Bacteriological Nomenclature* (Bacteriological Code) was drafted during the Third International Microbiological Congress (New York, 1939) and approved for publication at the Fourth International Microbiological Congress (Copenhagen, 1947). The unambiguous application of names assured by the preservation of nomenclatural types in type culture collections was among the essential problems addressed in this Bacteriological Code (Buchanan et al. 1948). The issue of allowing living cultures of bacteria as types decisively led to the split with the microbiologists (Buchanan 1959) and the publication of the *International Code of Nomenclature of Bacteria and Viruses* in 1953 and later elimination of bacterial names (other than cyanobacteria which continue to be treated as algae for nomenclatural purposes) from the ICBN in 1975 (ICBN Leningrad Code; Stafleu et al. 1978).

Mycologists had campaigned for living cultures to be allowed as nomenclatural types from IMC2 in 1977 (van Warmelo 1979), but with no success. The proposal made by Hawksworth (1993) dealt among others with the problem of cultures as nomenclatural types and included two recommendations and one example. That example was approved for inclusion at the International Botanical Congress (IBC) in Tokyo in 1993. As the result, the ICBN Tokyo Code (Greuter et al. 1994) was the first to refer to a preservation technique that had to be applied for the type specimen (Art 8.2, Ex. 1, ‘*permanently preserved in a metabolically inactive state*’), in addition to the statement that *type specimens of names of taxa must be preserved permanently and cannot be living plants or cultures*. This and later ICBN and ICNafp *Codes* used *Candida populi* to exemplify properly deposited type material.

*“8.2. Type specimens of names of taxa must be preserved permanently and cannot be living plants or cultures. Ex. 1. The strain CBS 7351 is acceptable as the type of the name* Candida populi *Hagler et al. (in Int. J. Syst. Bacteriol. 39: 98. 1989) because it is permanently preserved in a metabolically inactive state by lyophilization.”*

The proposal only succeeded in getting approval because of the ‘*metabolically inactive state*’, and this became the first official recognition of permanent preservation techniques (e.g. cryopreservation and freeze-drying) for the type material of fungi. Another part of the proposal became Recommendation 8B.2 (ICBN Tokyo Code; Greuter et al. 1994), which specified cultures made from the metabolically inactive type.

*“In cases where the type of a name is a culture permanently preserved in a metabolically inactive state (see Art. 8 Ex. 6), any living isolates obtained from that should be referred to as “ex-type” (ex typo), “ex-holotype” (ex holotypo), “ex-isotype” (ex isotypo), etc., in order to make it clear they are derived from the type but are not themselves the nomenclatural type.”*

Although recognizing the need for studying and preserving cultures of fungi, Gams et al. (1998) argued that cryopreservation did not guarantee that the material remains completely unchanged and proposed to limit the applicability of Art 8.2, Ex. 1 to certain groups of fungi, including ascomycetous and basidiomycetous yeasts. That proposal also provided an overview of measures to safeguard the type material in culture collections. Many of these measures are routinely used by collections nowadays (see below). The proposal by Gams et al. (1998) was considered at the IBC in Saint Louis in 1999, but was rejected. However, a part of that proposal was incorporated in a modified Art. 8.4 which replaced Art. 8.2 of the ICBN Tokyo Code (Greuter et al. 1994).

*“Type specimens of names of taxa must be preserved permanently and may not be living plants or cultures. However, cultures of fungi and algae, if preserved in a metabolically inactive state (e.g. by lyophilization or deep-freezing), are acceptable as types.”*

The requirement to specify the preservation method in the place of valid publication was first introduced in the ICNafp Shenzhen Code (Turland et al. 2018) and applies only to species described on or after 1 January 2019 (Art. 40.8).

*“For the name of a new species or infraspecific taxon published on or after 1 January 2019 of which the type is a culture, the protologue must include a statement that the culture is preserved in a metabolically inactive state.”*

### Dead or alive

The requirement of the ICNafp for a clear designation and safe preservation of the type material is reasonable. The problem arises from how to interpret the term ‘living cultures’. Yeasts are recovered from a wide range of sources (Péter et al. 2017), often as colonies growing on culture media together with filamentous fungi and bacteria (Kurtzman et al. 2011). With the exception of non-culturable species, such as *Macrorhabdus ornithogaster* and *Pneumocystis* species, yeasts are commonly isolated, purified, and studied on culture media under controlled laboratory conditions to observe, for example, the lifecycle, as well as physiological and molecular properties. Therefore, in the descriptions of yeast taxa, the type material is referred to by several expressions, such as strain, type strain, or (type) culture, to reflect the fact that the material under study is ultimately a culture. The words ‘strain’ or ‘culture’ tell us the yeast was cultured and studied with living material comprising many cells that may not all be genetically identical. This does not imply that culture collections maintain type material as continuous living cultures that continuously accumulate mutations. The ICNafp Shenzhen Code refers to both ‘living’ and ‘active’ cultures under the Art. 8.4 (also in the glossary; Turland et al. 2018). Preserved yeast cultures are ‘living’, but not ‘metabolically active’.

The ICBN Tokyo Code (Greuter et al. 1994) was the first to give a legal status to the nomenclatural type in the form of a ‘*permanently preserved in a metabolically inactive state*’ culture. The example of *C. populi* was introduced in the *Code* to protect retroactively the names of yeasts and other fungi described from cultures (over 400 species described since 1958, Gams et al. 1998) where the method of preservation was not specified in the papers, but where it was not known if there were air-dried cultures or microscopic slide preparations, but there were cultures deposited in collections using cryopreservation and/or lyophilization (Hawksworth 1993). The use of cryopreservation by repositories for strain preservation also ensures that cultures are metabolically inactive and, thus, can be viewed as type specimens according to the Art. 8.4 of the ICNafp (see below). The published description of *C. populi* does not specify the preservation technique used to maintain the type strain, which was one reason it was chosen – to make the point that the method need not actually be cited in the work. However, the wording of Art. 8.4, Ex. 12 in the *Code* (ICNafp Shenzhen Code, Table 1) may have caused confusion as the original text of the description of *Candida populi* (Hagler et al. 1989) does not differ from a typically employed text used in the description of most new species of yeasts. However, this is why that example was used to show that specific reference to metabolically inactive cultures was not a requirement. That was also the case with the names considered as invalid by Kijpornyongpan and Aime (2017), *J. angkorensis* (Sipiczki and Kajdacsi 2009) and *Sympodiomycopsis lanaiensis* (Mahdi et al. 2008).

The original (English) description of *Candida populi* (Hagler et al. 1989) reads as follows:

*“The type strain of C. populi, strain UCD-FST 68-675B, was isolated from an exudate of Populus tremuloides (trembling aspen) at Delta Junction, Ala. This strain has been deposited in the collection of the Yeast Division of the Centraalbureau voor Schimmelcultures, Delft, The Netherlands, as strain CBS 7351 and in the American Type Culture Collection, Rockville, Md., as strain ATCC 64933.”*

Article 8.4 of ICNafp (Shenzhen and Melbourne Codes) permits deposition of a culture for algae and fungi if they are preserved in a metabolically inactive state. The article does not say anything about the need to specify the preservation method but states the following:

*“Type specimens of names of taxa must be preserved permanently and may not be living organisms or cultures. Nevertheless (written ‘However’ in the ICNafp Melbourne Code), cultures of algae and fungi, if preserved in a metabolically inactive state (e.g. by lyophilization or deep-freezing to remain alive in that inactive state), are acceptable as types.”*

It was known at that time that the practice of both CBS and ATCC was to preserve filamentous fungi and yeast strains in a metabolically inactive state, which made them acceptable; the *C. populi* example therefore conformed to the ICBN *Code* then in operation. Since then, many culture collections adopted and improved long-term cryopreservation techniques (see below).

### Collection practices

One of the main purposes of culture collections is the preservation of microbial diversity. Culture collections generally preserve their material in a metabolically inactive state (WFCC guidelines, http://www.wfcc.info/guidelines/) and they have increased their skills and quality standards following ISO 9001, ISO 17025, and NF S 96–900 certifications (Boundy-Mills et al. 2016). Some European culture collections, such as BCCM, CBS, CECT, CIRM, and DSMZ, have acquired the status of Biological Resource Centers in the past decade. All certified and some accredited culture collections also consider biosafety and biosecurity issues by following published management guidelines (Boundy-Mills et al. 2016). Many culture collections have adopted a professional management system, standard operational procedures, and databases to keep records, in order to ensure that their holdings will be properly preserved as viable and pure cultures and that the method for the identification of yeasts to the species level is sound, which is essential for safe and patent deposits (Boundy-Mills et al. 2016). Collection certificates are the documents that provide evidence of the availability of the type material. Typically, such certificates state that material has been received, checked, and preserved in the open collection following internal quality standards. Although a species description usually does not define the method of strain preservation, such information can be retrieved from the specified collection and certificate of deposit.

Although this may appear intuitive and trivial to zymologists, it is important to emphasize to mycologists in general that yeasts are commonly isolated and purified on solid or liquid culture media. Their initial characterization includes macro- and micro-morphological examination, a bank of biochemical tests and molecular phylogenetic information with partial sequences of the ribosomal RNA gene(s), other genomic regions, or increasingly entire genomes (Libkind et al. 2020). Potential novel species are, in addition, biochemically and physiologically characterized, with standard techniques (Kurtzman et al. 2011). In many cases, multiple strains with similar characteristics are compared (in the case of *Candida populi* Hagler et al. 1989, 23 strains were analyzed), and a single strain is selected by the authors as the type strain. A carefully identified and characterized isolate is deposited in one or more culture collections as the type strain, as defined in Table 2. The culture collections control the authenticity of the received material and perform long-term preservation of the culture, following common procedures and standards (Smith and Ryan 2012). The importance of quality-controlled preservation of cultures has been acknowledged in the *Code*, and recommendation to deposit the type material in a “*reputable culture collection*” in the ICBN Berlin Code (Greuter et al. 1988) was succeeded by the wording that specifically referred to “*genetic resource collections*” in Rec. 8.1 of the ICNafp Shenzhen Code (Turland et al. 2018; Table 2).

### The more, the better

The importance of a reliable deposition of algae and fungal types has been acknowledged in the ICNafp (Shenzhen Code; Turland et al. 2018). In order to ensure the preservation of the type material, the ICBN Berlin Code (Greuter et al. 1988) recommended (Rec. 9A.1) deposition in a reputable culture collection:

*“Whenever practicable a living culture should be prepared from the holotype material of the name of a newly described taxon of fungi or algae and deposited in a reputable culture collection.”*

This recommendation was modified (ICBN Tokyo Code; Greuter et al. 1994) following a proposal of Hawksworth (1993) to ensure that the material is safeguarded in at least two collections; this is now Rec. 8.1 of the ICNafp Shenzhen Code (Turland et al. 2018):

*“Whenever practicable a living culture should be prepared from the holotype material of the name of a newly described taxon of fungi or algae and deposited in at least two institutional culture or genetic resource collections.”*

Following that recommendation, researchers have used subcultures of type strains (holotypes) preserved in culture collections to revise the taxonomy of yeasts, tracing them back to the material cited in original species descriptions. In addition to the literature, a collection certificate and a collection catalog (nowadays usually a dynamic online database) attests to the existence, authenticity and availability of the type material. A unique combination of a collection acronym and accession number allows searching of cultures (see ICNafp Shenzhen Code; Turland et al. 2018: Art. 40.7, Note 4 and Rec. 9B.1 in Table 2). Some collections provide additional information regarding strain authentication, such as accession and identification date, and published quality control sequences. This creates a transparent system in which each user is able to access the strain, its history (e.g. isolator, identifier, and depositor) and associated metadata through a collection catalog.

To ensure that the type strain(s) remain(s) available even when a culture collection discontinues its activity or loses the strain, a practice of deposition in at least two culture collections was also introduced by the *International Code of Nomenclature of Prokaryotes* (ICNP; see Tindall et al. 2010; Parker et al. 2019). Accordingly, leading microbiological journals, including *IJSEM* and *Antonie van Leeuwenhoek*, modified their instructions for authors requiring, among others, a collection certificate to prove the deposition of the type material. Originally applied only to prokaryotes, the requirement of the ICNP affected the description of yeast taxa such that journals began to prompt (and control) the deposition of proposed type strains of yeasts in culture collections. Some journals recommended a certain collection (usually CBS) in their instructions to authors (e.g. Lachance 2020). Moreover, most journal editors require the deposition of reference materials in more than one culture collection (preferably in different countries) to ensure improved accessibility to the user community and also mitigate the risk in case of an accident in one collection that could result in the loss of reference material. However, as a direct result of the aforementioned recommendation (i.e. deposition in at least two collections), yeast taxonomists found themselves in conflict with ICNafp Art. 40.7 (see below). Note that the example of *Candida populi* provided in ICNafp Art. 8.4 Ex. 12 (Shenzhen Code; Turland et al. 2018) also cites deposition in CBS and ATCC collections, as strains CBS 7351 and ATCC 64933, respectively.

Yeast colonies may contain genetically different cell types due to mutations that may result in polymorphisms, and subcultures of the same strain may differ from each other. The use of well-established protocols and at least two different modes of preservation contribute to long-term preservation, with the aims of minimizing genetic and phenotypic drift and reducing strain-identity errors. When strains are stored in culture collections, they can be repeatedly reactivated (e.g. using preservation batches from different years), controlled, and replaced, which is not the case for dried fungarium specimens. The likelihood of the occurrence of errors, such as strain substitution, when depositing to more than one culture collection by the original depositor is minimal, and when they do happen, they can be easily corrected (Fell et al. 2007; Duarte et al. 2012). For instance, the so-called type strain [in fact ex-type strain] of *Sporothrix flocculosa* CBS 167.88 (later distributed to other collections and stored as strains JCM 10321, NBRC 10875), was initially considered to be an ascomycete (Traquair et al. 1988), later found to be a member of the *Ustilaginomycotina* (Boekhout 1995), and was recently erroneously transferred to the genus *Anthracocystis* (Piątek et al. 2015) because the wrong strain was used. This could be traced and corrected because the correct living material was additionally [to the dried holotype DAOM 196992] preserved in the University of Alberta Microfungus Collection as strain UAMH 5743. The substitution of strains was of importance as *Sporothrix flocculosa* was used to develop the commercial biological control agent Sporodex™ (e.g. Paulitz and Bélanger 2001; Mimee et al. 2005). Due to the presence of multiple deposits, this error could be corrected (R. Bélanger and T. Boekhout, unpubl. data). Another example is strain CBS 9136, an ex-type strain of *Mrakia curviuscula*, which was found not to be identical to two representatives of the original culture (Oz-358), namely KBP Y-3618 (holotype, freeze-dried) and another ex-type VKM Y-2953 (Babjeva et al. 2002). Thanks to the practice of depositing ex-type strains in two culture collections, the taxonomic position of the species could be corrected (Kachalkin et al. 2019). Had a single dead fungarium specimen been used as holotype of a yeast species, such a correction would have been impractical, to say the least. In another example, the status of *Saccharomycodes sinensis* (now *Yueomyces sinensis*) was restored after it was discovered that an incorrect strain was deposited in the NRRL collection (Wang et al. 2015a). The availability of ex-type strains from additional collections allowed this error to be corrected.

A unique combination of a collection acronym and accession number ultimately defines the holotype of a taxon and/or its duplicates (ICNafp Shenzhen Code; Turland et al. 2018: Art. 40.7, Note 4 and Rec. 9B.1). When a mistake occurs, a correction mechanism should be in place. The designation of a holotype made in the protologue can be corrected for errors in data on locality, date, collector, collecting number, herbarium (including fungarium) code, specimen identifier, or citation of an illustration (ICNafp Shenzhen Code Art. 9.2). Such corrections can occur without the need for excessive renaming and alterations of authorship (which invariably result in the creation of multiple confusing species identifiers). Contrary to this, omissions of information required under Art. 40.6, 40.7 and 40.8 are not correctable under the present wording of the ICNafp Code (Turland et al. 2018). This restriction makes it difficult or even impossible to provide benign corrections of specimen identifiers.

## CONFUSING TERMINOLOGY

The ICNafp Shenzhen Code permits the deposition of a metabolically inactive culture as a type (glossary; Turland et al. 2018: Arts 8.4, 40.8), while Rec. 8B.2 distinguishes the original material and cultures (ex-type, ex-holotype, ex-isotype) derived from it (Tables 1 and 2). It is important to mention that, in the case of yeast taxa, a deposition is almost always made with an original living culture that has not already been transformed into a [metabolically inactive state by means of cryopreservation and/or freeze-drying] specimen in the sense of the ICNafp Art. 8.4 (Shenzhen Code; Turland et al. 2018). Because the nomenclatural type may not be a living culture (Art 8.4), the material becomes a type (specimen) only after being permanently placed (non-viable) in a fungarium, or (viable but metabolically inactive) in a culture collection.

The yeast taxonomic literature abounds with examples of confusion in the terminology used in protologues.

1. The original culture is often designated as “type strain”, which does not fully conform with Art. 40.6 (ICNafp Shenzhen Code; Turland et al. 2018), which states:
  *“For the name of a new taxon at the rank of genus or below published on or after 1 January 1990, indication of the type must include one of the words “typus” or “holotypus”, or its abbreviation, or its equivalent in a modern language.”*

However, the holotype of *Candida populi* was indicated as type strain in Art. 8.4 Ex. 12 (ICNafp Shenzhen Code; Turland et al. 2018).

(2) Although commonly cited in protologues as ex-types, metabolically inactive strains preserved in [one or several] culture collections are not ex-types because they are not kept as living cultures and because they are not derived from metabolically inactive cultures. In fact, they are duplicates [produced by sub-culturing] of the original culture [before it has been preserved in a metabolically inactive state] and are isotypes (Art. 9.5 ICNafp Shenzhen Code). An isotype is defined as *“any duplicate of the holotype; it is always a specimen”*. Isotypes can be deposited or preserved in different institutions, i.e. culture collections (Art. 40.7 ICNafp Shenzhen Code). Isotypes have been used by mycologists to safeguard the holotype material in culture collections and fungaria (e.g. Toome et al. 2013; Tanaka and Honda 2017; Sugiyama et al. 2018). The ICNafp Shenzhen Code (and earlier versions alike) does not require a certain wording and the use of holotype and isotypes has varied between studies. The description of *Meredithblackwellia eburnea* includes both the holotype (NRRL Y-48821) and two isotypes, CBS 12589 and ATCC MYA-4884 (Toome et al. 2013). However, in this case the isotypes are duplicates of the holotype obtained by subculturing the same original in the laboratory of the depositor. Recently, the type material of the smut fungus *Macalpinomyces spermophorus* was [correctly] designated in the description as isotypes, i.e. duplicates of the holotype, BPI 166627 and H.U.V. 10,545 (Tanaka and Honda 2017). In our opinion, the statement that lists the collections where the isotypes were deposited, each with a unique collection identifier (e.g. CBS 10918 = CCY 88–1-1, Sipiczki and Kajdacsi 2009) conforms with the [intention of the] *Code* because an exact wording for the designation of isotypes is not required by the ICNafp Shenzhen Code.
(3) The preservation of strains in culture collections follows several important steps which ensure that the material is properly safeguarded by means of cryopreservation and/or freeze-drying, and often with an additional backup. A culture is preserved with several technical replicates, such as cryotubes, beads, straws, or vials. All replicates receive the same collection identifier. When needed, metabolically inactive cultures can be reactivated (cultured) to produce ex-type cultures for dispatch. As a consequence, there is [usually] no single lyophilized tube or freezing vial that contains the holotype. Strictly speaking, when the single lyophilized tube or freezing vial that contains the holotype, if there is one, is opened, the holotype is lost. Such a loss does not usually occur in culture collections as organisms are preserved in several replicates and batches. We therefore suggest that one should not overinterpret the complexity of the ICNafp Code regarding the availability and authenticity of the type material preserved in a metabolically inactive state. Article 8.3 (ICNafp Shenzhen Code) allows more than one preparation of the specimen as long as the parts are clearly labelled as being part of that same specimen, or bear a single, original label in common. Thus, all properly labelled cryopreserved and/or freeze-dried technical replicates of the same culture constitute the type specimen.
(4) It is important to note that when both Latin and English diagnoses or descriptions were provided, both are part of the protologue (Table 2). These texts are not always identical and should be carefully examined before considering a name invalid. The *Code* requires the clearly designated holotype linked to a name. The name will be still validly published even when the diagnoses contain errors or when published illustrations are attributed to wrong species (Lentz and Hawksworth 1971).
(5) In our opinion the requirement of Art. 40.8 (ICNafp Shenzhen Code) is excessive because the information about preservation techniques used by culture collections is provided in the CCINFO database in Art. 40.7 Note 4 and therefore implicit in the mention of a collection in the description. Since the first application of cryopreservation techniques for fungi, methodologies have been improved and optimized for the vast majority of cultivable taxonomic groups, such as *Ascomycota*, *Basidiomycota*, *Blastocladiomycota*, *Chytridiomycota*, *Monoblepharidomycota*, and *Mucoromycota* (Smith and Ryan 2012; Simmons et al. 2020). Collections that utilize cryopreservation for their holdings systematically apply these techniques to all their cultures. Unlike the requested statement, a certificate issued by a culture collection is a more reliable source of information about preservation techniques.

### Our comment on Art 8.4

To date, culture collections and larger Biological Resource Centers (BRCs) routinely use an array of techniques to preserve microbial resources as freeze-dried (lyophilized powders) or frozen (liquid nitrogen, − 140 °C and − 80 °C freezers) material (Smith and Ryan 2012). Information on how the major collections preserve their cultures is publicly available through the Culture Collections Information Worldwide (CCINFO) database of the World Federation for Culture Collections (WFCC) and World Data Centre for Microorganisms (WDCM) (see http://www.wfcc.info/ccinfo/). The specification of a preservation method was adopted in the ICNafp Shenzhen Code and applies only to species described on or after 1 January 2019 (Art. 40.8). Thus, in a case like that of Kijpornyongpan and Aime (2017), we believe that the authors overinterpreted Art. 8.4 (ICNafp 2011 Melbourne Code; McNeill et al. 2012) in stating that the description of the genus *Jaminaea* and the two species *J. angkorensis* and *J. lanaiensis* were invalid according to Art. 8.4 of the ICNafp Melbourne Code. Both species were deposited in the CBS collection of the Westerdijk Fungal Biodiversity Institute (Utrecht, The Netherlands) where they are maintained as both lyophilized powders and deep-frozen in liquid nitrogen; hence, the types were preserved in a metabolically inactive state. They are available in the collection catalog, including the associated quality control data. The respective publications (Sipiczki and Kajdacsi 2009; Mahdi et al. 2008) and the CBS collection catalog records each document the depositions. The claim that the two strains in CBS were not preserved in a metabolically inactive state was not substantiated by any evidence.

### Our opinion on Art 40.7

In our opinion, cases like that of the genus *Jaminaea* (Kijpornyongpan and Aime 2017), *Acaromyces*, *Farysizyma*, and *Meira* (Denchev and Denchev 2021) where the authors stated that original descriptions of the genera were invalid, but for a different reason, namely because the protologue cited more than one culture collection for the deposition of the type material (Art. 40.7), are valid to some extent. The practice of deposition of the reference material in several culture collections followed recommendations of previous versions of the *Code* (from ICBN Tokyo Code; Greuter et al. 1994; also Rec. 8B.1 ICNafp Shenzhen Code; Turland et al. 2018), a practice that was also promoted by journal policies (see above). Names that may be invalid according to Art. 40.7 are probably the most numerous and easy to correct by indicating one [of several listed] collection strain as the metabolically inactive holotype (Denchev and Denchev 2021). It is important to mention that the latest edition of the taxonomic compendium *The Yeasts, a Taxonomic Study* (*TYTS*, Kurtzman et al. 2011), an effective publication in the sense of the ICNafp Art. 29.1 (Shenzhen Code; Turland et al. 2018), provided curated records of all yeast species accepted at the time of publication. A single strain deposited in a culture collection was indicated as a type strain, thereby providing an univocal indication of holotypes for the majority of invalid yeast names, including those validated by Kijpornyongpan and Aime (2017) and Denchev and Denchev (2021). One can debate on whether *TYTS* qualifies as a correction and was conforming at the time of publication with provisions of the ICBN Vienna Code (McNeill et al. 2006), but it is not in debate that this taxonomic compendium clearly indicated nomenclatural types for all yeast species accepted by the community. Thus, the recent validations in genera *Acaromyces*, *Jaminaea*, and *Meira* simply repeat the indication of nomenclatural types, which has been effectively published by taxonomic experts in *TYTS* 10 years ago thanks to the original material that was still extant, authenticated, and traceable.

Recent validations attempted to provide corrections, but this did not bring more clarity as validations only slightly adjusted the wording to meet the formal requirements leaving several important questions open, some of which are summarized in the ICNafp Rec. 9A (Shenzhen Code; Turland et al. 2018). While indicating the holotype in a validation, it is essential to prove that it is authentic and is indeed preserved in a metabolically inactive state as it was indicated in the original publication. Do authors of a validation present the evidence, such as in the form of culture collection certificates? Who and how to decide which strain to select as the holotype and which duplicate strains will remain as isotypes or ex-types? In our opinion, this nearly impossible task can only be achieved by contacting authors and collection curators. If validations leave the responsibility for correct indication of strain numbers, authenticity, deposition type, etc., to the authors of the original publications, the same can be achieved by a correction under the Art. 9.2.

A single holotype ensures unequivocal association between the material and the name, but the single holotype is useful only when it is authentic and correct and agrees with the described properties. A formally valid name has little use if it is not associated with authentic, traceable reference material; see for example the discussion on the genus *Hansenula* (Kurtzman 2011; Daniel et al. 2012). Therefore, the community of scientists working with yeasts gradually developed a system to preserve strains representing nomenclature types in several collections and authentication thereof by collections curators. We also think that a workable mechanism of deposition authentication like that established in the ICNP 2008 revision (Parker et al. 2019) could be considered for adoption in the ICNafp to handle properly fungi that are predominantly or exclusively known from cultures. Rule 30 (3b) of the ICNP regulating valid publication of a species name states the following:

*“As of 1 January 2001, the description of a new species, or new combinations previously represented by viable cultures must include the designation of a type strain (see Rule 18a), and a viable culture of that strain must be deposited in at least two publicly accessible culture collections in different countries from which subcultures must be available. The designations allotted to the strain by the culture collections should be quoted in the published description. Evidence must be presented that the cultures are present, viable, and available at the time of publication.”*

Importantly, this wording covers both the deposition and availability of the material. The latter is ensured by deposition in at least two culture collections (see also ICNafp Shenzhen Code, Rec. 8B.1; Turland et al. 2018), the viability of the material, and its availability for distribution.

In our view, the number of name changes due to incorrect typification has to be kept to a minimum, and should ideally be zero. Communities of taxonomists that study microorganisms should be aware that taxonomy is, to a large extent, a service to non-taxonomist users, who repeatedly express dismay at the growing number of name changes that accompany new methodological developments. Yet another round of name changes, arising from capricious nomenclatural dictates, will afflict the user community, be it clinical, biotechnological, food-associated, or intellectual property (Yutin and Galperin 2013; Lawson et al. 2016; Warnock 2019), and adversely affect large databases, such as GenBank, patent databases, and clinical guidelines that need to implement name changes. Routine taxonomic changes can rapidly incite debates around naming species of medical importance, highlighting the responsibility of scientists to patients who may experience health risks due to the wrong treatment being given because of name change (Kidd et al. 2020).

An example is the case of *Candida auris*. This species was described based on a single isolate obtained from the external ear canal of a 70-year-old woman who was an inpatient at a Japanese hospital (Satoh et al. 2009). The type strain, JCM 15448, was also deposited as CBS 10913 and DSM 21092, without referring to holotype, ex-types, or isotypes, with the wording “*Typus stirps JCM15448*” in the Latin protologue. Whether or not this description effectively conformed to the intent of the *Code* (the nomenclatural type is a strain), it would only be considered invalid by a nomenclature purist. This yeast species and its name are of the highest importance, as witnessed by a for *Candida auris*, which returned > 553,000 hits in Google search, 12,700 records in Google Scholar and 643 publications listed in PubMed as of 5 February 2021. *Candida auris* ranks at the top of the field of clinical mycology due to its multidrug resistance. Many clinical warnings have been published by public health authorities, such as the CDC [https://www.cdc.gov/drugresistance/solutions-initiative/stories/cdc-response-to-global-threat.html], or the PAHO [https://www.paho.org/hq/dmdocuments/2016/2016-oct-3-phe-candida-auris-epi-alert.pdf], the WHO, and the ECDC [https://ecdc.europa.eu/sites/portal/files/documents/RRA-Candida-auris-European-Union-countries-first-update.pdf]. A proposal to change this name based on the misunderstanding of ambiguity of the indication of the holotype would result in disastrous confusion for clinical mycology, microbiology, epidemiology, infectiology, and the patients that suffer from an infection by *C. auris*. The importance of taxonomic descriptions, recognition, and delimitation of species, as well as preservation of the type material in microbiology, is beyond all question. Nevertheless, it is important to remember that the impact of taxonomic changes can be broad ranging and, thus, limiting unnecessary changes is highly desirable.

## A possible solution

MycoBank lists approximately 300 names of yeasts as invalid according to Art. 40.7 (as of 25 August 2020) of which two thirds (including names currently considered to be synonyms) are names of yeasts for which authentic type material is available. We are aware of other species accounts that do not conform to the *Code* but are listed as validly published. Nomenclatural repositories kindly provide access to published names of fungi, including information on the current name, holotype (specimen or ex-type), literature, and taxonomic status, and whether the name is valid and legitimate. Although taxonomic repositories are managed by experts, errors can occur at any time. For example, *Bullera miyagiana*, the basionym of *Sugitazyma miyagiana*, which is the type species of the genus *Sugitazyma*, was listed as invalid according to Art. 40.1 and 40.7 (ICNafp Melbourne Code; McNeill et al. 2012) in MycoBank and Index Fungorum (search on 1 April 2019). However, the description of this yeast includes the holotype [original culture] 7586-ss-2 (conforming with Art. 40, Rec. 40A) deposited in the [single] JCM culture collection as JCM 7536 (conforming with Art. 40.7): *“Holotypus: Isolatus ex Abiete firma, Tohoku Univ., Sendai, Miyagi Pref., Japonia, 1986, R. J. Bandoni, JCM 7536 (originaliter ut 7586-ss-2) conservatur in collectionibus culturarum quas “Japan Collection of Microorganisms, Wako, Saitama” sustentat.”.* The nomenclatural curators of MycoBank (Konstanze Bensch) and Index Fungorum (Paul Kirk) accepted our arguments and modified the records of *Bullera miyagiana* accordingly. Importantly, the message provided by curators of nomenclatural repositories has a strong effect on the user community. However, the opinion published by a nomenclature repository should not be used as an incentive to scrutinize all existing species descriptions in the hope of identifying trivial mistakes of wording, with the side-effect of rendering useless the past and present work and practices of taxonomic sub-commissions, such as the Yeasts Working Group of the ICTF affiliated with ICY.

Recently, several authors have validated fungal names by retaining the original names and authorship, making reference to the original description for the diagnosis, and selecting only one collection for the deposition of the type material (e.g. Fonseca et al. 2008; Sun et al. 2011; Crous et al. 2019). However, following this practice would still require a designation of a holotype, new publication, and record in an electronic nomenclatural repository, Fungal Names, Index Fungorum, or MycoBank, because the ICNafp Art. 9.2 (Shenzhen Code; Turland et al. 2018) does not allow omissions according to Art. 40.6–40.8 (see below). It is also important to note that, when a name was published (before 1st January 1990, Art. 40.7) without a holotype (or if the holotype is lost or destroyed), the nomenclatural type should be designated as a lectotype (Table 2). Lectotypes have to be registered and the number for that cited as for a new taxon published on or after 1 January 2019 in accordance with Art. F5.4 (ICNafp Shenzhen Code; Turland et al. 2018). Another important point is that the validated names would date only from the time when the validation and/or lectotypification was made. They are not validated retroactively. An attempt to correct or complete the information on the holotype without a validation and/or lectotypification unintentionally increases the number of records for a species (though only one record will be valid at the end), making it difficult to understand for non-taxonomist and taxonomist users alike.

The ICNafp Shenzhen Code Chapter F (San Juan Chapter F, May et al. 2019) provides a mechanism to protect (or reject, if needed) currently accepted names in a list of protected names (Art. F.2 and F.7). The mechanism of protecting names in lists is rather simple and should be used to stabilize the nomenclature of fungi in the future. Once reviewed and approved by the Nomenclature Committee for Fungi (NCF) and the General Committee of International Botanical Congress (IBC), these names will be listed with their types and treated as conserved against any competing listed or unlisted synonyms. The ICY and the Yeasts Working Group of the ICTF should produce lists of protected names for currently recognized species to address taxonomic changes and fix their typifications; this would also safeguard them against being displaced by long-forgotten names that might be resurrected through new typifications. We believe that the database www.theyeasts.org (Boekhout et al. 2020) will be a useful source of information in the future about correct and presently used scientific names of yeasts.

## Possible proposals

Below we summarize nomenclatural proposals that, in our opinion, will improve the stability of fungal names, in particular by protecting those of yeasts for which the reference material can be traced back to the original isolate. The final text of the proposals will be released after incorporation of suggestions from taxonomy experts of the ICTF. The discussion of the problem of potentially invalid names of yeasts was timely brought in the yeast community by the proposal made by Kijpornyongpan and Aime (2017). Here, we illuminate provisions of the ICNafp (Shenzhen Code; Turland et al. 2018), which are rather impractical and unsustainable for fungi known from cultures. We hope that subsequent debates within the mycological community, including researchers working with cultivable fungi other than yeasts, will shape the ideas presented below into solid proposals that are likely to be implemented in the ICNafp in the future. In our opinion, it is important to elaborate in the *Code* two mechanisms of indication of nomenclatural types, such as (a) inviable holotype and viable ex-types, and (b) viable holotype (and at least one isotype) strains and their progenies. Our proposals aim at the creation of flexible but credible and transparent mechanisms. We do not intend to introduce further restrictions, so that taxonomists working with fungi with pleomorphic lifecycles may be able to choose either way of typification.

### Proposals to clarify the kind of material that represents the nomenclatural type

These proposals aim to streamline the present practice of publication of new taxa at the rank of species. As it has been demonstrated above with several examples, there is confusion and inconsistency regarding the phraseology used to designate the nomenclatural type. In our opinion, it is necessary to clarify what is meant by “type material” in the protologue (Art. 40.6). We propose to include “holotype strain” and “isotype strain” in addition to the words “type” and “holotype” in Art. 40.6. The need to designate strains representing the nomenclatural type is of importance for many culturable fungi. With this proposal, we intend to establish a transparent system to document what kind of material has been studied and how it is preserved. This change would not result in any conflict with the present practice because the use of metabolically inactive cultures as nomenclatural types is permitted by Articles 8.4, 40.7, and 40.8. In particular, Art. 40.8 requires a statement regarding the deposition of a culture in inactive state. The expression “type culture” would not contradict Art. 8.4. The word “culture” is already used in the ICNafp Shenzhen Code and its glossary. However, using in the protologue the word “strain” and not “culture” is advantageous to clearly distinguish cultures preserved in a culture collection. In the *International Code of Nomenclature of Prokaryotes* (Parker et al. 2019), the term “strain” in the designation of the nomenclatural type is not restricted to the strain bearing the culture collection number mentioned in the valid publication but refers to any culture knowingly derived from the original strain (Table 3; ICNP Rule 18c). This reflects collection practices, such as deposition of type strains in several culture collections and subsequent exchanges.

**Table 3:** Relevant provisions of the ICNafp (Turland et al. 2018) and ICNP (Parker et al. 2019) regulating the designation and preservation of the nomenclatural type

ICNafp	ICNP
**Article 8.1** The type (holotype, lectotype, or neotype) of a name of a species or infraspecific taxon is either a single specimen conserved in one herbarium or other collection or institution, or a published or unpublished illustration (but see Art. 8.5; see also Art. 40.4, 40.5, and Art. 40 Ex. 6). **Article 8.2** For the purpose of typification a specimen is a gathering, or part of a gathering, of a single species or infraspecific taxon, disregarding admixtures (see Art. 9.14). It may consist of a single organism, parts of one or several organisms, or of multiple small organisms. A specimen is usually mounted on a single herbarium sheet or in an equivalent preparation, such as a box, packet, jar, or microscope slide. **Article 8.4** Type specimens of names of taxa must be preserved permanently and may not be living organisms or cultures. Nevertheless, cultures of algae and fungi, if preserved in a metabolically inactive state (e.g. by lyophilization or deep-freezing to remain alive in that inactive state), are acceptable as types (see also Art. 40.8). **Article 40.5** For the purpose of Art. 40.1, the type of a name of a new species or infraspecific taxon of microscopic algae or microfungi (fossils excepted: see Art. 8.5) may be an effectively published illustration if there are technical difficulties of specimen preservation or if it is impossible to preserve a specimen that would show the features attributed to the taxon by the author of the name. **Article 40.6** For the name of a new taxon at the rank of genus or below published on or after 1 January 1990, indication of the type must include one of the words “typus” or “holotypus”, or its abbreviation, or its equivalent in a modern language (see also Rec. 40A.1 and 40A.4). But in the case of the name of a monotypic (as defined in Art. 38.6) new genus or subdivision of a genus with the simultaneously published name of a new species, indication of the type of the species name is sufficient. **Article 40.7** For the name of a new species or infraspecific taxon published on or after 1 January 1990 of which the type is a specimen or unpublished illustration, the single herbarium, collection, or institution in which the type is conserved must be specified (see also Rec. 40A.5 and 40A.6). Note 4. Specification of the herbarium, collection, or institution may be made in an abbreviated form, e.g. as given in *Index Herbariorum* or in the *World Directory of Collections of Cultures of Microorganisms*. **Article 40.8** For the name of a new species or infraspecific taxon published on or after 1 January 2019 of which the type is a culture, the protologue must include a statement that the culture is preserved in a metabolically inactive state. **Recommendation 40A.1** The indication of the nomenclatural type should immediately follow the description or diagnosis and should include the Latin word “typus” or “holotypus”. **Recommendation 40A.4** Details of the type specimen of the name of a new species or infraspecific taxon should be published in the Latin alphabet. **Recommendation 40A.5** Specification of the herbarium, collection, or institution of deposition should be followed by any available number permanently and unambiguously identifying the holotype specimen. **Recommendation 40A.6** Citation of the herbarium, collection, or institution of deposition should use one of the standards mentioned in Art. 40 Note 4 or, when those standards give no abbreviated form, should be given in full with the location.	**Rule 18a** Whenever possible, the type of a species or subspecies is a designated strain. The type strain is made up of living cultures of an organism, which are descended from a strain designated as the nomenclatural type. The strain should have been maintained in pure culture and should agree closely to its characters with those in the original description (see Chapter 4C). The type strain may be designated in various ways (see Rules 18b, 18c, and 18d). 1. Until 31 December 2000, for a species (or subspecies) which has not so far been maintained in laboratory cultures or for which a type does not exist, a description, preserved specimen, or illustration (see also Rule 18f) may serve as the type. *Example*: Non-cultivated, *Oscillospira guilliermondii* Chatton and Perard 1913. 2. As from 1 January 2001, a description, preserved (non-viable) specimen, or illustration may not serve as the type.
**Article 40.1** Publication on or after 1 January 1958 of the name of a new taxon at the rank of genus or below is valid only when the type of the name is indicated (see Art. 7–10; but see Art. H.9 Note 1 for the names of certain hybrids).	**Rule 16** The type of a taxon must be designated by the author at the time the name of the taxon is published in the IJSEM (see Rules 15, 18a, b, f, 20a-c, 21a, 22, 27 (3)). Note. Authors who intend to publish the name in the IJSEM with reference to a previous effectively published description under Rule 27 (2) are advised also to designate the type when publishing that description. Note. If a previous effective publication does not designate a type then the type must be designated at the time of valid publication in IJSEM, in accordance with the Rules of this Code.
**Article 9.1** A holotype of a name of a species or infraspecific taxon is the one specimen or illustration (but see Art. 40.4) either (a) indicated by the author(s) as the nomenclatural type or (b) used by the author(s) when no type was indicated. As long as the holotype is extant, it fixes the application of the name concerned (but see Art. 9.15).	**Rule 18b** Designation by original author If the author in the effective publication of the name of a species or subspecies definitely designated a type strain, then this strain shall be accepted as the type strain and may be referred to as the holotype.
**Article 9.8** A neotype is a specimen or illustration selected to serve as nomenclatural type if no original material exists, or as long as it is missing (see also Art. 9.16 and 9.19(c)). **Article 9.11** If the name of a species or infraspecific taxon was published without a holotype (Art. 9.1), or when the holotype or previously designated lectotype has been lost or destroyed, or when the material designated as type is found to belong to more than one taxon, a lectotype or, if permissible (Art. 9.8), a neotype as a substitute for it may be designated (see also Art. 9.16). **Article 9.13** If no original material is extant or as long as it is missing, a neotype may be selected. A lectotype always takes precedence over a neotype, except as provided by Art. 9.16 and 9.19(c). **Article 9.16** When a holotype or a previously designated lectotype has been lost or destroyed and it can be shown that all the other original material differs taxonomically from the lost or destroyed type, a neotype may be selected to preserve the usage established by the previous typification (see also Art. 9.18). **Article 9.18** A neotype selected under Art. 9.16 may be superseded if it can be shown to differ taxonomically from the holotype or lectotype that it replaced. **Article 9.19** The author who first designates (Art. 7.10, 7.11, and F.5.4) a lectotype or a neotype in conformity with Art. 9.11–9.13 must be followed, but that choice is superseded if (a) the holotype or, in the case of a neotype, any of the original material is found to exist; the choice may also be superseded if it can be shown that (b) it is contrary to Art. 9.14 or (c) it is in serious conflict with the protologue, in which case an element that is not in conflict with the protologue is to be chosen; a lectotype may only be superseded by a non-conflicting element of the original material, if such exists; if none exists it may be superseded by a neotype.	**Rule 18c Designation as neotype** If a strain on which the original description was based cannot be found, a neotype strain may be proposed. A neotype strain must be proposed (proposed neotype) in the IJSEM, together with citation of the author(s) of the name, a description or reference to an effectively published description, and a record of the permanently established culture collection(s) where the strain is deposited (see also Note 1 to Rule 24a). The author should show that a careful search for the strains used in the original description has been made and that none of them can be found. The author should also demonstrate that the proposed neotype agrees closely with the description given by the original author. The neotype becomes established (established neotype) 2 years after the date of its publication in the IJSEM, provided that there are no objections, which must be referred within the first year of the publication of the neotype to the Judicial Commission for consideration. Note. The term “strain” refers to the culture or subcultures of it, described in the original description. This is not restricted to the strain bearing the culture collection number mentioned in the valid publication, but refers to any culture knowingly derived from the original strain.
	**Rule 18f** If a description or illustration constitutes, or a dead preserved specimen has been designated the type of a species (Rule 18a(1)) and later a strain of this species is cultivated, then the type strain may be designated by the person who isolated the strain or by a subsequent author. This type strain shall then replace the description, illustration or preserved specimen as the nomenclatural type. The designation of a type strain in this manner must be published in the IJSEM, the authorship and date of priority of publication being determined by the effective and valid publication of the name by the original authors (Rule 24b).
**Recommendation 8B.1** Whenever practicable a living culture should be prepared from the holotype material of the name of a newly described taxon of algae or fungi and deposited in at least two institutional culture or genetic resource collections. (Such action does not obviate the requirement for a holotype specimen under Art. 8.4.) **Recommendation 8B.2** In cases where the type of a name is a culture permanently preserved in a metabolically inactive state (see Art. 8.4), any living isolates obtained from it should be referred to as “ex-type” (ex typo), “ex-holotype” (ex holotypo), “ex-isotype” (ex isotypo), etc., in order to make it clear they are derived from the type but are not themselves the nomenclatural type.	**Rule 30** 3.b: As of 1 January 2001, the description of a new species, or new combinations previously represented by viable cultures must include the designation of a type strain (see Rule 18a), and a viable culture of that strain must be deposited in at least two publicly accessible culture collections in different countries from which subcultures must be available. The designations allotted to the strain by the culture collections should be quoted in the published description. Evidence must be presented that the cultures are present, viable, and available at the time of publication. Note. In exceptional cases, such as organisms requiring specialized facilities (e.g. Risk Group/Biological Safety Level 3, high pressure requirements, etc.), exceptions may be made to this Rule. Exceptions will be considered on an individual basis by a committee consisting of the Chairman of the ICSP, the Chairman of the Judicial Commission and the Editor of the IJSEM. Exceptions will be made known at the time of publication. 4. Organisms deposited in such a fashion that access is restricted, such as safe deposits or strains deposited solely for current patent purposes, may not serve as type strains.

The holotype fixes the application of the name (Art. 9.1). A set of rules and recommendations were developed to ensure that the material is safely preserved. They include the recommendation to preserve living cultures of fungi and algae in at least two institutional culture or genetic resource collections (Art. 8, Rec. 8B.1). The practice of depositing the type material in several culture collections and subsequent exchanges between collections is essential to safeguard the material, as we have also mentioned above. However, there has been considerable confusion regarding the specification of the type material deposited in several culture collections. There is an urgent need to provide clear recommendations and examples to avoid mistakes and misinterpretation in the future. As we indicated, duplicates of original type cultures that are preserved in a metabolically inactive manner in culture collections are in fact isotypes, not ex-types. Therefore, the words “isotype” and “strain” can be added to the protologue to clearly indicate the deposition of any viable metabolically inactive duplicate culture in another repository. Provisions of the ICNafp Shenzhen Code already provide mechanisms to distinguish the original material, holotype and its duplicates, at the time of description and its subsequent progenies, for example as ex-holotype, ex-isotype. Mentioning explicitly duplicates of the holotype, isotypes, and ex-type cultures is an important consideration in the case if the holotype is lost. This change would not result in any conflict with the present practice but will clarify the origin of the material and rules applied to it. We also suggest developing clear wording to harmonize future publications regarding the use of isotypes in protologues.

*Specific proposals*

(1A) A proposal to modify Article 40.6 to permit the use of the expressions “holotype strain” and “isotype strain” to designate the type material in the protologue.

(1B) A proposal to add the following note, example and recommendation to Art. 40.7: Metabolically inactive material produced from subcultures of the original material deposited by authors for the purpose of the valid description in collections different from the one in which holotype is deposited, are isotypes (according to Arts. 8.4, 9.5, 40.8), not ex-type cultures and not different specimens and, thus, conform with Art. 40.7. This does not apply to strain exchange between culture collections after publication of the original description, which should be cited as ex-holotype or ex-isotype strains.

(1C) A proposal to modify Recommendation 8B.2 of Art. 8 in accordance with Art. 40.7.

We consider that some of these proposals can be also implemented in Chapter F of the ICNafp Shenzhen Code, for example in Art. F5 dealing with the registration of names and nomenclatural acts (May et al. 2019). Specifically, Chapter F Arts. F.5.2, F.5.6 - F.5.8 governing the registration of identifiers of names and nomenclatural types. The minimum elements of information that must be accessioned for registration of new scientific names already include elements required by Art. 40.7 (Art. F5.2). This provision can be further expanded and structured to include the information about the nomenclatural type.

### Proposal to allow amendments to the information required under Articles 40.6, 40.7, and 40.8 in Art. 9.2

With the examples given above, we have demonstrated the need for a transparent system to track the availability and authenticity of type material. Therefore, it is important to ensure that records referring to that material in repositories remain up-to-date. To achieve this aim:

(2A) Art. 9.2 should be modified to allow omission related to Art. 40.6–40.8.

In a recent example, Zhao et al. (2019) described *Tremella basidiomaticola* and correctly specified the type specimen (*holotype strain: CGMCC 2.5724*^*T*^) in the China General Microbiological Culture Collection Center (CGMCC) and its subcultures in the CBS collection. Both repositories are listed by CCINFO. However, the authors did not include a statement about the preservation techniques used in the China General Microbiological Culture Collection Center, thus making the name invalid according to Art. 40.8 ICNafp Shenzhen Code. However, the record of the collection undoubtedly indicates that CGMCC collection uses cryopreservation for storage (http://www.wfcc.info/ccinfo/collection/by_id/550), and so this information could be easily corrected, possibly in the already existing identifier (Art. F5.1, F5.2, F5.4) without introducing a new identifier (see above). Kachalkin et al. (2019) attempted to correct the protologue, erroneously referring to Art. 9.2 ICNafp Shenzhen Code. As a result, this correction was made improperly and the name *T. basidiomaticola* remained invalid until Li et al. (2020) made the correct validation.

In our opinion, the present wording of Art. 9.2 creates unnecessary complications to make nomenclatural corrections of records related to the indication of the type material and the form of preservation. Alternatively, a similar mechanism of correction of information about nomenclature types could find its place in the Art. F.5 of the *Code*. Article F5.2 lists the minimum elements of information that must be accessioned by author(s) of scientific names and those elements required for valid publication (May et al. 2019). Requirement of Art. 40.8 should be included in Art. F5.2 as this directly concerns the indication of the nomenclatural type and, thus, valid publication (see the discussion above). A transparent system to track the availability and authenticity of type material could be adopted in the Art. F5 and achieved with electronic nomenclature repositories.

### Proposal to clarify the availability of the type material represented by a metabolically inactive culture in culture collections under Art. 8.4

The ICNafp Shenzhen Code recommends the deposition of cultures representing the holotype in at least two culture collections to ensure that the nomenclatural type is properly preserved. Yeasts are commonly isolated, purified, and studied on culture media under controlled laboratory conditions to observe, for example, the life-cycle, as well as physiological and molecular properties. Therefore, the viability of the nomenclatural type is absolutely essential to taxonomic and other studies (see the discussion above). In the case when the culture representing a nomenclatural type is lost or inviable, it should be replaced with a lectotype [strain] or neotype [strain] in accordance with provisions of Art. 9 and Art. F.5.

Seifert and Rossman (2010) discussed formal requirements and best practices for the publication of descriptions of new fungal species, including availability of type specimens. There is a growing concern related to various national and international regulations associated with the implementation of regulations related to the Nagoya Protocol (NP) supplementary agreement to the UN Convention on Biological Diversity (CBD), which was ratified by 120 nations (https://absch.cbd.int/). The NP recognizes the importance of biodiversity assessments *without removing specimens from their habitats* (https://www.cbd.int/gti/taxonomy.shtml). Phenotype-based research, such as *morphological analysis*, normally would also not amount to utilization under national legal frameworks, such as EU Regulation No. 511/2014. Unfortunately, phenotype-based research without removing specimens from their habitats is not possible for many fungi, including yeasts. As a result, nearly all microbiological and mycological research that depends on environmentally obtained organisms or samples must follow the national CBD and NP legislation where and when access (sampling) took place (reviewed in Yurkov et al. 2019). In some cases, “access” to the material is extended to the material in culture collections and fungaria alike. When “access” is determined as the date the material has been acquired from an ex-situ collection (not the date of sampling from the environment), the national regulation becomes retroactive.

The unavailability of specimens and national restrictions were mentioned among important obstacles to taxonomic research (Seifert and Rossman 2010), including cross-border exchange and loan of specimens (Prathapan et al. 2018). Because each country defines procedures and requirements to access resources by implementing a national legislation, conditions of access to the material at the time of deposition have to be made clear.

At present, ICNafp Shenzhen Code Art. 8.4 requires availability (*Type specimens of names of taxa must be preserved permanently*) but not [unrestricted] accessibility of the material. This brings the scientific community to the problem that any type that is not accessible under such legislation is not legally available for future scientific studies. The ICNP Rule 30 (Table 3) specifically mentions that restricted strains, such as safe deposits or strains deposited solely for current patent purposes, may not serve as type strains. It is meaningful to include a similar note in the INCafp. The *Code* Art. 8.4 and Rec. 8B.1 requires availability of the nomenclatural type and recommends its deposition in culture collections. However, viability of the nomenclatural type stored in a metabolically inactive state is not required by the ICNafp Shenzhen Code. Although this brings a certain degree of nomenclatural stability, dead specimens do not allow study and verification of important characters of most fungi, such as physiological properties, lifecycle, and secondary metabolites. Names of many culturable fungi for which no viable nomenclatural type is available cannot be verified. It is important to elaborate mechanisms for deposition of fungi that are predominantly or exclusively known from cultures. These provisions must include a mechanism to replace dead or inviable nomenclatural types, for example similar to those in ICNP Rule 18c (Table 3). Although there is a substantial overlap between provisions of the two *Codes* regarding the designation of the neotype (Table 3), mechanisms of the ICNafp Art. 9 (Table 3) should apply to both (a) strains (see above) and (b) their viability (see ICNP Rule 18c, 18f; Table 3). An inviable strain representing a nomenclatural type qualifies as its loss and need for a replacement.

*Specific proposals*

(3A) Add the following note to Art. 8.4 that when a viable holotype [strain] or a previously designated lectotype has been lost or destroyed, a viable neotype [strain] preserved in a metabolically inactive state may be selected in accordance with provisions of the Art. 9.

(3B) Add a recommendation to Art. 8.4 that whenever a specimen or a metabolically inactive preserved culture is deposited for the purpose of the typification of a new species, a statement on the availability of and access to the material should be submitted with the manuscript.

(3C) Add a recommendation to Art. 8.4 that strains with restricted access, such as safe deposits or strains deposited solely for current patent purposes, cannot qualify as a holotype strain unless a special material transfer agreement permits access to it for taxonomic purposes.

## Data Availability

Data sharing is not applicable to this article as no datasets were generated or analysed specifically for this purpose.

## ABBREVIATIONS

ATCC: American Type Culture Collection (USA)
BCCM: Belgian Coordinated Collections of Microorganisms
BRC: Biological Resource Center
CBD: UN Convention on Biological Diversity
CBS: The Centraalbureau voor Schimmelcultures, presently the Westerdijk Fungal Biodiversity Institute (the Netherlands)
CCINFO: Culture Collections Information Worldwide database
WFCC: World Federation for Culture Collections
CCY: Culture Collection of Yeasts (Slovakia)
CDC: Centers for Disease Control and Prevention (USA)
CGMCC: China General Microbiological Culture Collection Center
CECT: Spanish Type Culture Collection
CIRM: International Center for Microbial Resources (France)
DAOM: Canadian National Mycological Herbarium
DNA: Deoxyribonucleic acid
DSMZ: German Collection of Microorganisms and Cell cultures
ECDC: European Centre for Disease Prevention and Control
IBC: International Botanical Congress
ICBN: The International Code of Botanical Nomenclature
ICNafp: The International Code of Nomenclature for Algae, Fungi, and Plants
ICNP: The International Code of Nomenclature of Prokaryotes
ICTF: The International Commission on the Taxonomy of Fungi
ICY: The International Commission on Yeasts
IJSEM: International Journal of Systematic and Evolutionary Microbiology
IMC: International Mycological Congresses
ISSY: International Specialised Symposium on Yeasts
ISO: International Organization for Standardization
JCM: Japan Collection of Microorganisms
KBP: Yeast collection of the Soil Biology Department in Lomonosov Moscow State University (Russia)
NBRC: National Biological Resource Centre of Japan
NCF: The Nomenclature Committee for Fungi
NP: Nagoya Protocol on Access to Genetic Resources and the Fair and Equitable Sharing of Benefits Arising from their Utilization, a supplementary agreement to the Convention on Biological Diversity
NRRL: Agricultural Research Service Culture Collection (USA)
PAHO: Pan American Health Organization
RNA: Ribonucleic acid
TYTS: The monographic book ‘The Yeasts: A taxonomic study’ published between 1952 and 2011
UAMH: University of Alberta Centre for Global Microfungal Biodiversity (Canada)
UCD-FST: Phaff Yeast Culture Collection (USA)
VKM: All-Russian Collection of Microorganisms
WDCM: World Data Centre for Microorganisms
WHO: World Health Organization
